# WSCD2 Expression: Its Relevance to Tumor-Infiltrating Immune Cells and Glioma Prognosis

**DOI:** 10.2174/0109298673282921240220101914

**Published:** 2024-02-26

**Authors:** Kaderya Abudusalam, Yan Xu, Pazilat Keyumu, Tong Cheng, Manyu Xu, Bing Lu, Pingping Sun, Kadierjiang Musha, Jianfei Huang

**Affiliations:** 1 Department of Clinical Biobank, Affiliated Hospital of Nantong University & Department of Pathology, Medical School of Nantong University, Nantong, 226001, Jiangsu, China;; 2 Translational Medicine Center, People's Hospital of Kizilsu Kirgiz Autonomous Prefecture, 845350, Xinjiang Autonomous Region, China

**Keywords:** WSCD2, immune cell, prognosis, glioma, infiltration, immunotherapy

## Abstract

**Background:**

Patients with glioma have limited treatment options and experience poor prognoses. Therefore, it is urgently needed to explore new diagnostic and therapeutic targets.

**Objective:**

This study aimed to investigate the relevance of WSC domain-containing 2 (WSCD2) expression to glioma, clinicopathological characteristics, tumor-infiltrating immune cells (TILs), and patient prognosis.

**Methods:**

We analyzed WSCD2 mRNA expression in glioma tissues and patient survival using the Gene Expression Profiling Interactive Analysis database. Furthermore, the relationship between the expressions of WSCD2 mRNA and TILs in gliomas was evaluated utilizing the Tumor Immune Estimation Resource database. Lastly, we employed multiplex immunohistochemistry to detect the protein expressions of WSCD2 and TILs in glioma tissues.

**Results:**

WSCD2 mRNA expression in glioma tissues was lower than that in tissues of benign brain disease. High WSCD2 mRNA expression was also significantly associated with a favorable outcome. Additionally, WSCD2 mRNA expression was correlated with TIL expression in glioma; however, no such relationship was detected between the protein expressions of WSCD2 and TILs in glioma tissues. Cox regression multivariate analysis and Kaplan-Meier survival analysis showed that WSCD2 expression in glioma tissues could be an independent prognostic factor.

**Conclusion:**

This study highlights the correlation between WSCD2 expression and TILs and demonstrates the prognostic significance of WSCD2 in glioma. Furthermore, our results suggest that WSCD2 may be a potential immunotherapy target in glioma.

## 1 INTRODUCTION

Gliomas of the brain are the most common type of primary human tumor, with an incidence rate of 4.67 to 7.73 per 100,000 person-years [1-3]. These tumors arise in the brain's glial cells, such as astrocytes or oligodendrocytes [4]. Glioma tissue comprises interacting tumor cells and stromal cells, including a small number of non-transformed cells, tumor-infiltrating lymphocytes (TILs), and other immune effector cells that compose the tumor immune microenvironment (TIME) [5-7]. According to glioma histology, molecular characteristics, and aggressiveness, the World Health Organization (WHO) categorizes gliomas with substantial malignancy from grade I to IV. Gliomas are also classified as low-grade gliomas (LGGs) and high- grade gliomas (HGGs) [2-8], wherein HGGs are considered malignant gliomas [9]. Based on the glioma malignancy grading, Grade IV glioblastomas (GBMs) are the most aggressive tumors [2, 10], accounting for approximately 55% of malignant gliomas [11] and having an average survival of <2 years after the final diagnosis [2, 12, 13].

Although recent advances have been made in glioma therapies combining surgery, chemotherapy, radiotherapy, and other immunotherapy, patients still have limited treatment options and experience poor prognoses [12-14]. One major reason for this scarce progress is the lack of effective antigen-specific anti-glioma antigen targets. The second reason is the complex TIME network [15], along with other factors, such as the blood-brain barrier [4]and energy metabolism [16]. Nevertheless, the Cancer Genome Atlas (TCGA) program has made large-scale genomic glioma datasets available to identify tumor subtypes and analyze tumor heterogeneity [2, 16, 17], which can help patients receive appropriate medical treatment. However, more clinical trials and clinicopathological research are urgently needed to utilize these existing mRNA data and clinical information to develop clinical applications [2, 18].

Using TCGA and Genotype-Tissue Expression (GTEx) datasets, we found that the mRNA expression of the WSC domain-containing 2 (WSCD2) gene was not only related to the prognosis of patients with glioma but also linked to immune infiltration in glioma tissues. The role of WSCD2, also named KIAA0789, has been addressed in only a few studies [19-21]. WSCD2 plays an essential function in hypertension [19], insulin secretion in human islets [21], and psychiatric disorders [20]. To the best of our knowledge, this is the first study on the relationship between WSCD2 and neoplasms.

Due to the unreliability of mRNA expression in predicting protein levels, complex biological samples exhibit no linear relationship between these two parameters [22-24]. To further investigate the association between the protein expressions of WSCD2 and TILs in the glioma TIME, we identified the expression characteristics of WSCD2 and TILs mRNA in the glioma data of TCGA and GTEx *via* web servers. Furthermore, we hypothesized the relationship between the protein expressions of WSCD2 and TILs with the clinical factors using multiplex immunohistochemistry (mIHC) in glioma tissue microarrays (TMAs). Our novel findings highlight the clinical significance of WSCD2 as a promising potential target for the application of glioma immunotherapy. This investigation aimed to reveal the relationship of WSCD2 expression to glioma, clinical features, TILs, and patient outcomes.

## 2 MATERIALS AND METHODS

### 2.1 Gene Expression Profiling Interactive Analysis (GEPIA)

We used GEPIA2 (http://gepia2.cancer-pku.cn/#index) to evaluate WSCD2 mRNA expression and patient survival in glioma. GEPIA2 is an online database for analyzing the RNA sequencing expression of 9,736 tumors and 8,587 benign tissues from TCGA/GTEx (https://gtexportal.org/home/) datasets for tumor/normal comparison [25]. In this panel, the gene classes exhibit the types of 60,498 genes. The difference in WSCD2 mRNA expression was compared by boxplots using GBM (n = 163), LGG (n = 518), and tissues of benign brain (n = 207) disease as variables. Additionally, the relationship between WSCD2 mRNA expression and the prognosis of patients with glioma was evaluated using the “survival curve” modules of GEPIA2.

### 2.2 Tumor Immune Estimation Resource (TIMER)

The TIMER database (https://cistrome.shinyapps.io/timer/) is a systematic resource for the integrated analysis of TILs in 32 tumor types and includes 10,897 samples from TCGA [26]. The freely available web server can robustly estimate multiple TILs, including T cells, B cells, neutrophils, macrophages, and dendritic cells. We analyzed the relationship of WSCD2 mRNA expression with the gene markers of TILs *via* the correlation modules of TIMER. Next, we submitted the WSCD2 gene in glioma and types of TILs, and the scatter plots showed the relevant associations between the gene expression and infiltrate estimation value.

### 2.3 Human Glioma Specimens and Clinical Features of the Patients

From January, 2012 to December, 2017, 157 formalin-fixed paraffin-embedded tissues from 146 patients with glioma and 11 with benign brain disease were obtained from the Department of Clinical Biobank, Affiliated Hospital of Nantong University. Representative tissue areas were re-reviewed and assembled into 2-mm TMAs using the Manual TMA system (Quick-Ray; UNITMA, Korea). The clinical information of the patients with glioma was acquired from their medical records, including gender, age, histological classification, and tumor grade according to the 2016 WHO Classification of Central Nervous Tumors. All the patients in the study had not undergone chemotherapy, radiation therapy, or immunotherapy before surgery. The 5-year overall survival was determined from the initial biopsy to death. The study protocol was approved by the Human Research Ethics Committee of the local hospital (2018-K020).

### 2.4 IHC and mIHC

The TMA slides were baked at 65ºC for 1 h, followed by dewaxing and hydration using xylene and alcohol. The protocol for IHC has been described previously [27]. Briefly, the TMA sections were incubated with anti-IDH1R132H antibody (1:10, DIA-H09, Dianova, Germany) at 4°C overnight and then incubated with a secondary antibody for 2 h at room temperature. Finally, these sections were developed with diaminobenzidine and hematoxylin.

For mIHC staining, we used the Opal™ 7-Color Manual IHC Kit (L810001KT, PerkinElmer, USA) based on the manufacturer's instructions. After rehydration, the slides were fixed for 20 min in 10% neutral buffered formalin. These slides were then heated using a microwave for 15 min in an AR6/AR9 buffer to retrieve the antigen. Next, the slides were incubated with the primary antibody as follows: anti-WSCD2 antibody (1:500, orb620966, Biorbyt, UK), anti-CD3 antibody (1:500, 85061S, CST, USA), anti-CD4 antibody (1:5000, ab133616, Abcam, UK), anti-CD8 antibody (1:2500, ab93278, Abcam), anti-CD20 antibody (1:1000, ab78237, Abcam), anti-CD56 antibody (1:200, 3576, CST, USA), anti-CD66b antibody (1:1000, arg66287, Airgobio, China), anti-CD68 antibody (1:500, 76437, CST, USA), anti-CD83 antibody (1:1000, Mab1774, R&D, USA), and anti-GFAP antibody (1:500, ab33922, Abcam). Lastly, Opal™ secondary-HRP (polymer HRP Ms+Rb) and DAPI (nuclear staining) staining were performed, as described previously [28].

### 2.5 Multispectral Imaging and Multiplex Analysis

All stained tissue cores in the TMA sections were scanned into digital images and quantitatively evaluated using inForm 2.4 (image analysis software) in a Vectra 3.0 Quantitative Pathology Imaging Research System (PerkinElmer, USA). The inForm 2.4 software automatically identifies the tissue types based on the training regions (tumor cells or benign brain disease cells/lymphocytes/blank area) provided by the user. Additionally, the inForm software distinguishes the cells and calculates consistent scoring judgments based on that quantification. The fluorescent signals from WSCD2 and other immune cells in each tissue core were automatically recognized and scored in a percentage format (number of positively stained cells/number of nuclei *100% of each tissue core). The cutoff point of the WSCD2 score was statistically different based on the overall survival *via* the X-tile software (http://www.tissuearray.org/rimmlab, Yale University) [29].

### 2.6 Statistical Analysis

The Student's *t*-test was adopted to compare the variance between the groups. The chi-square test was used to determine the statistical significance between WSCD2 expression and clinical characteristics. Univariate and multivariate analyses using the Cox model were carried out to identify the clinical prognostic factors in patient survival. Survival analysis was performed using the Kaplan-Meier estimator and a log-rank test. The Spearman correlation method was applied to analyze the relationship between WSCD2 expression and the immune cells. All data were analyzed using the SPSS 20.0 statistical software package (SPSS Inc., Chicago, USA). A *p*-value <0.05 was deemed statistically significant. An *R*-value (correlation statistic) >0.2 was considered significant.

## 3 RESULTS

### 3.1 WSCD2 mRNA Expression and Patient Survival Analysis in Glioma *via* GEPIA2

**Fig. (1):**
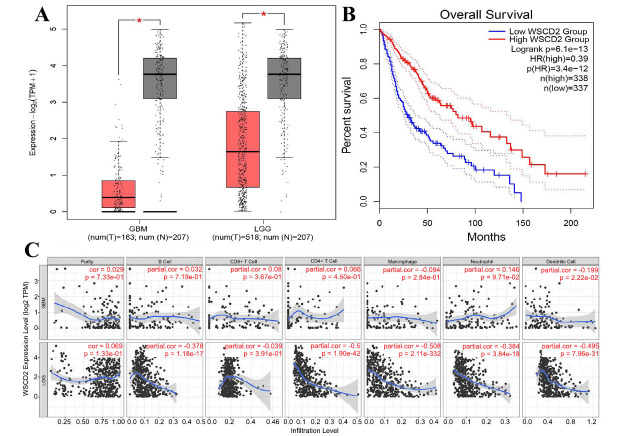
GEPIA analysis of WSCD2 mRNA expression differences and survival outcomes. (**A**): The different expressions of WSCD2 mRNA in normal brain tissues (gray color), LGG (red color), and GBM (red color). (**B**): The survival curve of WSCD2 mRNA expression in glioma patients (red line: high WSCD2 mRNA expression and blue line: low WSCD2 mRNA expression). (**C**): The relationship between WSCD2 mRNA expression and six types of TILs (B cells, T cells, macrophages, neutrophils, and dendritic cells).

Based on TCGA database and GTEx data, GEPIA2 revealed that WSCD2 mRNA expression in glioma tissues was lower than that in benign brain tissues (log2FC < 1, *p* < 0.01) (Fig. **1A**). Furthermore, considering the median WSCD2 mRNA expression as the group cutoff point, we also found that a high WSCD2 mRNA expression was significantly related to a favorable prognosis in patients with glioma. Fig. (**1B**) shows the Kaplan-Meier overall survival curve generated *via* GEPIA2 (*p* < 0.001).

### 3.2 Association between the Expressions of WSCD2 mRNA and TILs

We investigated the relationship between WSCD2 and the prominent immune infiltrates. Using the TIMER database, WSCD2 mRNA expression in LGG and GBM, respectively, was found to correlate with the expressions of B cells (*r* = 0.032, *p* > 0.05; *r* = −0.378, *p* < 0.01), CD8^+^ T cells (r = 0.08, *p* > 0.05; *r* = 0.039, *p* > 0.05), CD4^+^ T cells (*r* = 0.066, *p* > 0.05; *r* = −0.57, *p* < 0.01), macrophages (*r* = −0.094, *p* > 0.05; *r* = −0.508, *p* < 0.01), neutrophils (*r* = 0.146, *p =* > 0.05; *r* = −0.384, *p* < 0.01), and dendritic cells (*r* = −0.199, *p* < 0.05; *r* = −0.495, *p* < 0.01) (Fig. **1C**). These results indicated a clear negative correlation between the expressions of WSCD2 mRNA and three types of TILs (CD4^+^ T cells, macrophages, and dendritic cells) in GBM tissues.

### 3.3 Correlation between the Aberrant Protein Expressions of WSCD2 and TILs in Glioma Tissues

**Fig. (2):**
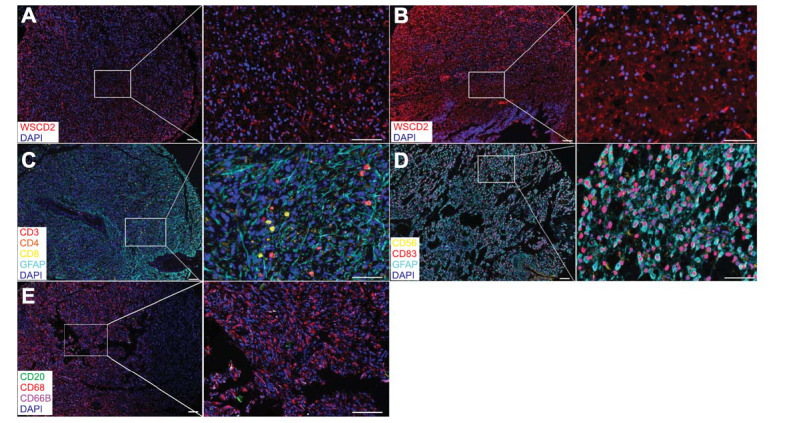
Representative fluorescence images showing WSCD2 and TIL subsets in TMA sections of glioma. (**A**, **B**): WSCD2 protein expression (red color) and DAPI (blue color) in glioma and non-tumor brain tissue. (**C**) CD3^+^ (red color), CD4^+^ (orange color), CD8^+^ (yellow color), GFAP+ (cyan-blue color), and DAPI (blue color) in glioma tissue. (**D**) CD56^+^ (yellow color), CD83^+^ (red color), GFAP+ (cyan-blue color), and DAPI (blue color) in glioma tissue. (**E**) CD20^+^ (green color), CD68^+^ (red color), CD66B^+^ (purple color) and DAPI (blue color) in glioma tissue.

The correlation between mRNA and protein expression levels is notably poor in biological samples [22-24]. Therefore, we used mIHC to investigate the protein expressions of WSCD2 and TILs in the TMA slides containing glioma and benign brain disease. WSCD2-positive staining was mainly localized in the cytoplasm of glioma and glial cells (Fig. **2**), as demonstrated by inform 2.4.. Furthermore, the level of WSCD2 protein in glioma tissues was lower than that in tissues of benign brain disease, with a significant difference (*t* = 2.104, *p =* 0.003) *via* the Student's *t*-test. Thus, our protein expression results were consistent with the results of WSCD2 mRNA expression according to the GEPIA tool.

To further evaluate the association between the protein expressions of WSCD2 and TILs in glioma TIME, we analyzed the score of the fluorescent biomarkers using Spearman’s correlation. The results demonstrated that WSCD2 protein expression was weakly correlated with the protein expressions of CD20^+^ B cells (*r* = 0.288, *p =* 0.001), CD4^+^ T cells (*r* = 0.231, *p =* 0.026), CD8^+^ T cells (*r* = 0.215, *p =* 0.038), CD68+ macrophages (*r* = 0.136, *p =* 0.116), CD66B+ neutrophils (*r* = 0.242, *p =* 0.005), and CD83+ dendritic cells (*r* = 0.201, *p =* 0.016) in glioma tissues (Fig. **2B**, **3A**-**E**). Therefore, these findings were not congruent with the mRNA expression results of the GEPIA tool.

### 3.4 Relationship between WSCD2 Protein Expression and Clinical Features of Glioma

The chi-square test was used to examine the relationship between WSCD2 protein expression and glioma clinical features. The cutoff point of the WSCD2 protein expression score was set to 60, according to the prognosis of the patients with glioma *via* X-tile software. Here, the cutoff value divided the glioma tissue samples into groups with high and low WSCD2 protein expressions. The results revealed that WSCD2 protein expression was strongly related to gender (χ2 = 4.820, *p =* 0.028) and tumor grade (χ2 = 12.837, *p =* 0.002) but not linked to age, histological classification, IDH1-R132H mutation status, and GFAP expression (Table **1**).

### 3.5 Association between the Protein Expressions of WSCD2 and TILs and Glioma Survival

**Fig. (3):**
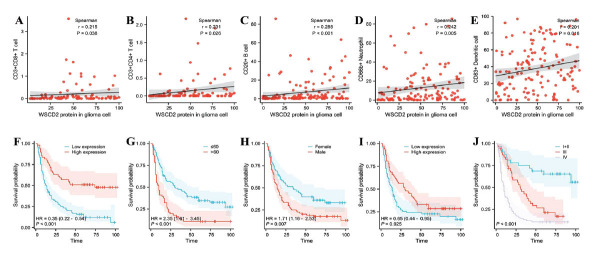
(**A** - **E**). The positive association of WSCD2 and TILs (CD8^+^T cells, CD4^+^T cells, CD20^+^ B cells, CD66B^+^ neutrophil, and CD83^+^ dendritic cells). (**F** - **J**). Kaplan-Meier curves and log-rank test, WSCD2 protein expression (high, red line; low: blue line), age (≤ 60 years: green line; > 60 years, red line), gender (female, red line; male, blue line), IDH1-R132H status (positive, red line; negative. blue line), and grade classification (I-II, red line; III. blue line; IV, green line).

Using univariate Cox analysis, we demonstrated the relationship between WSCD2 protein expression (hazard ratio [HR] = 0.351, *p* < 0.001), age (HR = 2.305, *p* < 0.001), gender (HR = 1.698, *p =* 0.008), IDH1-R132H status (HR = 0.653, *p =* 0.027), CD68^+^ macrophages (HR = 1.833, *p =* 0.003), and tumor grade (HR = 1.890, *p* < 0.001) and the lifespan of the patients with glioma. Additionally, these factors were evaluated using multivariate Cox analysis. The results indicated that WSCD2 protein expression (HR = 0.464, *p =* 0.002), age (HR = 1.851, *p =* 0.005), gender (HR = 1.643, *p =* 0.022), IDH1-R132H mutation status (HR = 0.629, *p =* 0.027), and tumor grade (HR = 1.513, *p =* 0.001) were independently correlated with the prognosis of patients with glioma (Table **2**). Furthermore, the Kaplan-Meier survival curves and log-rank test showed that these factors served as independent prognostic factors of glioma survival (Figs. **3F**-**J**).

## 4 DISCUSSION

Gliomas, including LGG and GBM, have high lethality, with the worst survival period of only a few months. Considering the lack of effective treatment for these patients, including the limited efficacy of various immunotherapies, identifying new immune-related targets for the clinical evaluation of gliomas is urgent. Although the genomic data in the public resources have provided the molecular features of gliomas, data on visible protein expression, location, and correlation analysis remains limited. To our knowledge, our study is the first to examine the relationship between the protein expressions of WSCD2 and TILs and the clinical features and prognosis of glioma.

In recent years, several studies have highlighted the importance of brain tumor immunology, which could influence the clinical treatment effect [30]. Many studies have reported the association between tumor-specific antigens and TILs with the TIME, as well as their relationship with glioma tissues [31, 32]. A small study has been initiated to investigate the microenvironmental landscape of TILs in gliomas and has already revealed part of the therapeutic mechanism [30, 33, 34]. Further understanding of the glioma TIME can help elucidate the immunotherapy mechanism.

In this study, we carried out bioinformatics analysis to examine the expressions of WSCD2 mRNA and TILs in glioma. WSCD2 mRNA expression in GBM was negatively correlated with the expressions of CD4^+^ T cells, macrophages, and dendritic cells. Using glioma TMA, mIHC, and multiplex analysis, we revealed critical insights into the localization and protein expression of WSCD2 and the abundance of TILs in glioma tissues. Our results demonstrated that WSCD2 protein expression has a weak positive correlation with the protein expressions of B cells, CD4^+^ T cells, CD8^+^ T cells, CD66B^+^ neutrophils, and CD83^+^ dendritic cells. These inconsistent results may be attributed to the expression of WSCD2 and TILs in complex biological stages. Macrophages are one of the main components of TILs in glioma [35]. Our univariate analysis of CD68^+^ macrophages in glioma tissues revealed that these macrophages were related to poor survival. These results suggest that glioma cells with low WSCD2 expression and different types of TILs could jointly promote tumor cell proliferation and immune escape. However, the interactive effect of these positively correlated cells in glioma tissues requires further research.

In the case of the differential WSCD2 expression in glioma tissues, it was observed that higher WSCD2 protein expression was strongly related to lower tumor grade and positive outcomes. Additionally, WSCD2 is involved in glucose metabolism [21], which, in turn, is related to cancer aggressiveness [36]. Thus, we suggest that WSCD2 may serve as a candidate biomarker of tumor suppression in glioma.

Our research has limitations. First, we did not conduct multi-center clinical samples for testing. Second, we did not use cellular and animal models to explore functions and mechanisms. Finally, this study could add to prospective clinical research.

## CONCLUSION

In summary, we identified that WSCD2 expression was related to TILs in tumor tissues and could serve as an independent prognostic factor for glioma. Furthermore, our study results indicated that WSCD2 is a potential target for immunotherapy in glioma.

## Figures and Tables

**Table 1:** Relationship between WSCD2 protein expression in glioma tissue and clinical features.

**Characteristic**		**WSCD2 Protein Expression**			
	**n**	**Low (%)**	**High (%)**	**Pearson χ2**	**P**
Total	146	97(66.43)	49(33.57)	-	-
Gender	-	-	-	4.820	0.028*
Male	84	62(73.81)	22(26.19)	-	-
Female	62	35(56.45)	27(43.55)	-	-
Age	-	-	-	1.015	0.314
≤60	90	57(63.33)	33(36.67)	-	-
>60	56	40(71.43)	16(28.57)	-	-
Histological classification	-	-	-	7.680	0.104
a: AA, AG, AGG	13	9(69.23)	4(30.77)	-	-
b: AO, AM, DA, OG	73	47(64.38)	26(35.62)	-	-
c: GM	30	25(83.33)	5(16.67)	-	-
d: DG	24	14(58.33)	10(41.67)	-	-
e: PA, PX, PMA	6	2	4	-	-
IDH-R132H mutation	-	-	-	2.107	0.147
yes	98	69(70.41)	29(29.95)	-	-
no	48	28(58.33)	20(41.67)	-	-
GFAP expression	-	-	-	2.874	0.090
Low	74	54(72.97)	20(27.03)	-	-
High	72	43(59.72)	29(40.28)	-	-
Tumor Grade	-	-	-	12.837	0.002*
I+II	22	14(43.75)	18(56.25)	-	-
III	39	24(61.54)	15(38.64)	-	-
IV	75	59(78.67)	16(21.33)	-	-

**Note: ** *p<0.05. a: anaplastic astrocytoma, anaplastic glioma, and anaplastic ganglioglioma. b: anaplastic oligoastrocytoma, astrocytoma, diffuse astrocytoma, and oligodendroglioma. c: glioblastoma multiforme. d: mixed glioma. e: pilocytic astrocytoma, pleomorphic xanthoastrocytoma, pilomyxoid astrocytoma.

**Table 2:** Univariate and multivariate analysis of survival in glioma patients.

-	**Univariate Analysis**				**Multivariate Analysis**			
	**HR**	**P**	**95% CI**		**HR**	**P**	**95% CI**	
WSCD2 expression High*vs* Low	0.351	<0.001*	0.223	0.552	0.464	0.002*	0.285	0.756
Age (years) ≤60 *vs.*>60	2.305	<0.001*	1.572	3.338	1.851	0.005*	1.206	2.841
Gender Male *vs.* Female	1.698	0.008*	1.149	2.511	1.643	0.022*	1.075	2.512
IDH1-R132H mutation yes*vs* no	0.653	0.027*	0.448	0.953	0.629	0.027*	0.417	0.949
Histological classification	0.983	0.643	0.895	1.080	-	-	-	-
CD20+B cell High*vs* Low	1.000	1.000	0.673	1.487	-	-	-	-
CD3+CD8+T cell High*vs* Low	1.213	1.213	0.758	0.758	-	-	-	-
CD3+CD4^+^ T cell High*vs* Low	1.041	0.869	0.646	1.676	-	-	-	-
CD68+ macrophage High*vs* Low	1.833	0.003*	1.232	2.727	1.452	0.079	0.958	2.200
CD66B+ neutrophil High*vs* Low	1.150	0.483	0.778	1.701	-	-	-	-
CD83+dendritic cell High*vs* Low	1.163	0.435	0.795	1.702	-	-	-	-
GFAP expression High*vs* Low	0.425	0.425	0.425	0.425	-	-	-	-
Tumor Grade I+II*vs* III*vs* IV	1.890	<0.001*	1.525	2.343	1.513	0.001*	1.188	1.929

**Note: ** *p<0.05. a: anaplastic astrocytoma, anaplastic glioma, and anaplastic ganglioglioma. b: anaplastic oligoastrocytoma, astrocytoma, diffuse astrocytoma, and oligodendroglioma. c: glioblastoma multiforme. d: mixed glioma.

## Data Availability

The data and supportive information are available within the article.

## LIST OF ABBREVIATIONS

WSCD2: WSC Domain-containing 2
TILs: Tumor-infiltrating Lymphocytes
TIME: Tumor Immune Microenvironment
WHO: World Health Organization
LGGs: Low-grade Gliomas
HGGs: High-grade Gliomas
GBMs: Glioblastomas
TCGA: The Cancer Genome Atlas
GTEx: Genotype-Tissue Expression
mIHC: Multiplex Immunohistochemistry
TMAs: Tissue Microarrays
GEPIA: Gene Expression Profiling Interactive Analysis
TIMER: Tumor Immune Estimation Resource

## STANDARDS OF REPORTING

STROBE guidelines were followed.
